# Associations of cannabis use, tobacco use, and incident anxiety, mood, and psychotic disorders: a systematic review and meta-analysis

**DOI:** 10.1017/S0033291724002587

**Published:** 2024-11

**Authors:** Chloe Burke, Tom P. Freeman, Hannah Sallis, Robyn E. Wootton, Annabel Burnley, Jonas Lange, Rachel Lees, Katherine Sawyer, Gemma M. J. Taylor

**Affiliations:** 1 Department of Psychology, University of Bath, Bath, UK; 2 School of Psychological Science, University of Bristol, Bristol, UK; 3 Centre for Academic Mental Health, Population Health Services, Bristol Medical School, University of Bristol, Bristol, UK; 4 Nic Waals Institute, Lovinsenberg Diaconical Hospital, Oslo, Norway

**Keywords:** anxiety disorders, cannabis, causal inference, confounding, epidemiology, mood disorders, psychotic disorders, systematic review, tobacco

## Abstract

**Background:**

Observational studies consistently report associations between tobacco use, cannabis use and mental illness. However, the extent to which this association reflects an increased risk of new-onset mental illness is unclear and may be biased by unmeasured confounding.

**Methods:**

A systematic review and meta-analysis (CRD42021243903). Electronic databases were searched until November 2022. Longitudinal studies in general population samples assessing tobacco and/or cannabis use and reporting the association (e.g. risk ratio [RR]) with incident anxiety, mood, or psychotic disorders were included. Estimates were combined using random-effects meta-analyses. Bias was explored using a modified Newcastle–Ottawa Scale, confounder matrix, *E*-values, and Doi plots.

**Results:**

Seventy-five studies were included. Tobacco use was associated with mood disorders (*K* = 43; RR: 1.39, 95% confidence interval [CI] 1.30–1.47), but not anxiety disorders (*K* = 7; RR: 1.21, 95% CI 0.87–1.68) and evidence for psychotic disorders was influenced by treatment of outliers (*K* = 4, RR: 3.45, 95% CI 2.63–4.53; *K* = 5, RR: 2.06, 95% CI 0.98–4.29). Cannabis use was associated with psychotic disorders (*K* = 4; RR: 3.19, 95% CI 2.07–4.90), but not mood (*K* = 7; RR: 1.31, 95% CI 0.92–1.86) or anxiety disorders (*K* = 7; RR: 1.10, 95% CI 0.99–1.22). Confounder matrices and *E*-values suggested potential overestimation of effects. Only 27% of studies were rated as high quality.

**Conclusions:**

Both substances were associated with psychotic disorders and tobacco use was associated with mood disorders. There was no clear evidence of an association between cannabis use and mood or anxiety disorders. Limited high-quality studies underscore the need for future research using robust causal inference approaches (e.g. evidence triangulation).

## Introduction

Tobacco and cannabis are two of the most commonly used recreational drugs worldwide. In 2019, approximately 1.14 billion adults globally had smoked tobacco regularly and an estimated 200 million people used cannabis in the last year (UNODC, 2021). Existing observational evidence demonstrates prospective associations between cannabis use, tobacco use, and mental illness; including depression, anxiety, and psychosis (e.g. Arango et al., 2021; Chaiton, Cohen, O'Loughlin, & Rehm, 2009; Chaplin et al., 2023; Esmaeelzadeh, Moraros, Thorpe, & Bird, 2018; Farooqui et al., 2022; Fluharty, Taylor, Grabski, & Munafò, 2017; Garey et al., 2020; Gobbi et al., 2019; Gurillo, Jauhar, Murray, & MacCabe, 2015; Hunter, Murray, Asher, & Leonardi-Bee, 2020; Lev-Ran et al., 2014; Luger, Suls, & Vander Weg, 2014; Marconi, Di Forti, Lewis, Murray, & Vassos, 2016; Moore et al., 2007; Myles et al., 2012; Robinson et al., 2023; Stevenson, Miller, Martin, Mohammadi, & Lawn, 2022; Zimmermann, Chong, Vechiu, & Papa, 2020). However, it remains unclear if the associations in question are causal or if they result from observational data biases (e.g. confounding, reverse causality; Hammerton & Munafò, 2021).

Confounding bias occurs when the effects of an exposure under study on a given outcome are ‘mixed in’ with effects of an additional factor, or set of factors, associated with the target exposure and outcome that results in a distortion of the true effect (Skelly, Dettori, & Brodt, 2012). Confounding bias can be reduced if appropriate controls are implemented (e.g. multivariable regression), but in practice it is difficult to measure all confounders and without error (Fewell, Davey Smith, & Sterne, 2007). Numerous reviews of these substances and mental illness highlight confounding bias as a key limitation (Chaplin et al., 2023; Garey et al., 2020; Gobbi et al., 2019; Gurillo et al., 2015; Hunter et al., 2020; Lev-Ran et al., 2014). However, no comprehensive assessment of the strength of potential confounding bias has been conducted.

In this review, confounding bias is evaluated using the confounder matrix (Petersen et al., 2022) and *E*-values (VanderWeele & Ding, 2017). The confounder matrix is an approach for defining and summarizing adequate confounding control in systematic reviews (Petersen et al., 2022). *E*-values are a quantitative approach to evaluate the sensitivity of estimates from an observational study to unmeasured confounding (D'Agostino McGowan, 2022; VanderWeele & Ding, 2017). Briefly, the *E*-value of an estimate represents the minimum strength of an association, on a risk ratio (RR) scale, that an unmeasured confounder would need to have with both the exposure and the outcome to reduce an observed effect estimate to the null (i.e. RR = 1), conditional on measured covariates (VanderWeele & Ding, 2017). Employed together, these tools provide a complementary and in-depth assessment of confounding bias.

A further difficulty is that co-use of cannabis and tobacco is highly common (Agrawal, Budney, & Lynskey, 2012; Gravely et al., 2020; Hindocha & McClure, 2020). Cannabis-tobacco co-use comprises concurrent use (i.e. use of both products in a pre-defined time period) and co-administration (i.e. simultaneous use within the same delivery method; Hindocha & McClure, 2020). Considering the high co-occurrence and associations with mental illness, there has been debate as to which, if any, has a more important role to play in the development of subsequent mental illness (Fergusson, Hall, Boden, & Horwood, 2015; Gage & Munafò, 2015). To our knowledge, few reviews examining psychological outcomes have considered evidence for *both* substances independently (Esmaeelzadeh et al., 2018) or jointly (Peters, Budney, & Carroll, 2012; Ramo, Liu, & Prochaska, 2012; Sabe, Zhao, & Kaiser, 2020). These reviews have limitations such as synthesizing predominantly cross-sectional studies (Peters et al., 2012; Ramo et al., 2012), focusing on specific geographic regions or clinical populations (Esmaeelzadeh et al., 2018; Sabe et al., 2020) and lack of quality and confounding assessment (Peters et al., 2012; Sabe et al., 2020).

As such, we aimed to synthesize longitudinal studies examining the association of cannabis and tobacco use with incident mental illness, with a focus on critically assessing biases that limit causal interpretation.

## Methods

We pre-registered our protocol on PROSPERO (CRD42021243903) and the Open Science Framework (https://osf.io/5t2pu/). We have followed PRISMA (Page et al., 2021) and MOOSE (Brooke, Schwartz, & Pawlik, 2021) reporting guidelines (online Supplementary eMethods 1), and described protocol changes in the online Supplementary materials (eMethods 2).

### Search strategy

We searched CINAHL, Embase, MEDLINE, PsycINFO, and ProQuest Dissertation and Theses from inception to November 2022. Searches were conducted using MeSH headings and text words relating to exposures, outcomes, and study design (online Supplementary eMethods 3). Supplementary searches were performed via forward and backward citation chasing, using the package *citationchaser* (Haddaway, Grainger, & Gray, 2021), and contact with experts for unpublished data. Screening was completed independently by two authors (CB and AB/RL/KS). Discrepancies were resolved through discussion among the reviewers, or a third reviewer where necessary (GT).

### Eligibility criteria

We included prospective longitudinal studies that (1) measured cannabis, tobacco, or co-use as an exposure, (2) used a ‘non-exposed’ comparator group, and (3) reported a relevant effect estimate (e.g. RR) and its variance, or necessary raw data. There were no restrictions on publication status, article language, or publication date. To minimize reverse causation bias we only included studies where participants with current indications (i.e. total incidence) and/or history (i.e. first incidence) of the outcome were excluded at baseline. Studies were also excluded if participants were selected on a specific health status (e.g. pregnancy), or other highly selected characteristics (e.g. incarcerated persons). Corresponding authors were contacted, where possible, to request missing effect estimates or information relating to study inclusion. Full details are given in Table 1.

**Table 1.** PECOS inclusion/exclusion criteria

Population	Broadly representative of general population Can be selected on basic demographics (e.g. age, sex, ethnicity, occupation), but not eligible if comprised of persons with particular health characteristics (e.g. chronic condition) or other clinically relevant factors (e.g. pregnancy, incarcerated adults, ‘high-risk’)
Exposure	Any measure of tobacco product use (e.g. current smoking, cigarettes per day [CPD]); excluding products not containing tobacco leaf (e.g. e-cigarettes) Any measure of recreational cannabis use (e.g. current use, ever use, lifetime frequency) Any measure of cannabis–tobacco co-use (e.g. concurrent use, co-administration)
Comparator	Eligible comparators are: (1) ‘non-exposed’ (e.g. never use, non-use, past year non-use, non-regular use); (2) other eligible exposures (e.g. *exp* = co-use, *ref* = tobacco use) Comparators of only lower frequency use groups (e.g. ⩾20 CPD *v*. ⩾10 CPD) or absence of problematic use (e.g. Cannabis Use Disorder [CUD] *v*. no CUD) are not eligible
Outcome	Can be any mood, anxiety, or psychotic disorder except for substance-induced disorders (e.g. cannabis-induced psychotic disorder) and outcomes that *only* measure Obsessive Compulsive Disorder (OCD) or Post Traumatic Stress Disorder (PTSD) Can be composite measures (e.g. ‘any mood disorder’) or specific conditions (e.g. depressive symptoms), but not eligible if combined across disorder groups (e.g. ‘any mental illness’) Must be a binary measure of an *incident* condition (i.e. analysis/study excluded participants with a current/lifetime history of condition) No limit on outcome measurement type, examples may include: self-rated scales, registry codes, interviews, self-reported diagnoses Must have raw data necessary for meta-analysis or pre-calculated effect estimate (e.g. odds ratio, risk ratio, hazard ratio, incident rate ratio)
Study design	Longitudinal design (cohort or nested case-control) and must collect and assess the exposure/outcome across multiple timepoints (i.e. retrospective recall not eligible) Studies without original data (e.g. systematic reviews, commentaries) are excluded, and case-reports, case-series, interventional studies, qualitative studies, animal studies, and in-vitro studies are excluded
Other	No limits on article publication status, year of publication, or language

### Data extraction

Standardized forms were used to extract study information by two independent reviewers (CB and JL). A modified Newcastle–Ottawa Scale (NOS) was used to evaluate study quality (Wells et al., 2013). The NOS evaluates studies across selection of study groups, comparability, and outcome ascertainment, awarding a total of nine stars. Studies were rated as ‘high’ quality if scoring: (i) maximum on items relating to comparability (i.e. confounding bias); (ii) maximum on items relating to attrition (i.e. selection bias); and (iii) only scoring less than one star on all other items (online Supplementary eMethods 4). A standardized assessment sheet was used (CB) and calibrated with a second-rater (JL) for ~20% of the included studies, and disagreements raised with a third reviewer (GT). If studies reported multiple estimates the following estimates were extracted: (i) longest follow-up length; (ii) highest frequency of use; and (iii) adjusted for most confounding variables.

### Data synthesis

We used the RR and the corresponding 95% confidence intervals (95% CIs) as the summary estimate. Included studies presented varied effect estimates and approach for conversion to RR is described in the online Supplementary materials (eMethods 5). Adjusted and unadjusted/minimally adjusted (i.e. age and sex) effect estimates were pooled separately. Considering study heterogeneity, random-effects meta-analysis using generic inverse variance approach was conducted. Between-study heterogeneity was explored through visual inspection of forest plots and tau-squared (*τ*^2^), and statistical inconsistency quantified using the *I*^2^ statistics (Higgins et al., 2020). Prediction Intervals (PIs) were also calculated, i.e. 95% range of true effect estimates to be expected in exchangeable studies (IntHout, Ioannidis, Rovers, & Goeman, 2016). Meta-analyses were conducted in R (v 4.4.1), using the ‘meta’ package (Schwarzer, 2022). Data and R scripts are available on GitHub (https://github.com/chloeeburke/tobcanmeta).

### Subgroup and sensitivity analyses

A combination of approaches was used to explore the impact of bias due to unmeasured confounding. The *E*-value represents the minimum strength of association, on an RR scale, an unmeasured confounder would need to have to fully explain a specific exposure–outcome association (i.e. fully reducing an RR to 1; VanderWeele & Ding, 2017). *E*-value calculation is described in the online Supplementary materials (eMethods 6). If the strength of suspected unmeasured confounding is weaker than indicated by the *E*-value, this suggests the exposure–outcome association is robust to unmeasured confounding (VanderWeele & Ding, 2017; VanderWeele, Ding, & Mathur, 2019). To assess the level of uncertainty associated with the effect, the *E*-value was also calculated for the CI closest to the null. There are no cut-offs for what constitutes a small or large *E*-value as it is context dependent, relative to the exposure, outcome, and measured covariates (VanderWeele et al., 2019). Therefore, we used a ‘confounder matrix’ assessment to establish measured covariates.

The confounder matrix is an approach to summarizing adequate confounding control in reviews of observational studies (Petersen et al., 2022), conducted in three steps: (1) expert consensus regarding necessary adjustment (e.g. constructs, measurement), (2) production of matrices to depict adjustment in each study, and (3) using assessment to inform quantitative synthesis (e.g. subgroup analyses). Based on a causal diagram (online Supplementary eMethods 7), studies in the primary meta-analyses were assessed on adjustment for seven constructs: co-use, other substance use, psychiatric comorbidity, socioeconomic status, sociodemographic factors, psychological factors, and other lifestyle factors. See online Supplementary eMethods 8 for description of constructs. The ‘E-Value’ online calculator (https://www.evalue-calculator.com/) and *metaconfoundr* package (Barrett, Petersen, & Trinquart, 2022) were used.

Where ⩾10 studies were available, sources of heterogeneity in the primary analyses were explored through pre-planned subgroup analyses and meta-regressions (Higgins et al., 2020). Additional exploratory analyses were conducted through (i) excluding outliers, defined as point estimates where the 95% CI lies outside the 95% CI of the pooled effect, and (ii) subgroup analysis by rating on the confounder matrix assessment.

Potential small-study effects, such as publication bias, were examined using Doi plots and the Luis Furuya-Kanamori (LFK) index (Furuya-Kanamori, Barendregt, & Doi, 2018). Doi plots visualize treatment effects on the *x*-axis and a normal rank-based *Z*-score on the *y*-axis. LFK indices less than ±1, greater than ±1 but less than ±2, or greater than ±2 were considered to represent no, minor, or major asymmetry, respectively (Furuya-Kanamori et al., 2018).

## Results

### Search results

Of the 27789 records screened, 486 studies were retained for full-text screening (online Supplementary eFig. 1). Studies excluded at full-text stage are available in the online Supplementary materials (eTable 1). We identified 75 studies for inclusion (Albers & Biener, 2002; Almeida et al., 2013; An & Xiang, 2015; Armstrong et al., 2017; Bakhshaie, Zvolensky, & Goodwin, 2015; Beutel et al., 2019; Bolstad et al., 2022; Borges, Benjet, Orozco, & Medina-Mora, 2018; Bots, Tijhuis, Giampaoli, Kromhout, & Nissinen, 2008; Breslau, Peterson, Schultz, Chilcoat, & Andreski, 1998; Brown, Lewinsohn, Seeley, & Wagner, 1996; Cabello et al., 2017; Chang, Pan, Kawachi, & Okereke, 2016; Chin, Wan, Choi, Chan, & Lam, 2016; Chireh & D'Arcy, 2019; Choi, Patten, Gillin, Kaplan, & Pierce, 1997; Clark et al., 2007; Cougle, Hakes, Macatee, Chavarria, & Zvolensky, 2015; Cuijpers, Smit, Ten Have, & De Graaf, 2007; Danielsson, Lundin, Agardh, Allebeck, & Forsell, 2016; do Nascimento et al., 2015; Feingold, Weiser, Rehm, & Lev-Ran, 2015, 2016; Flensborg-Madsen et al., 2011; Fonseca et al., 2022; Ford et al., 1998; Gage et al., 2015; Gentile, Bianco, Nordström, & Nordström, 2021; Goodman & Capitman, 2000; Goodwin et al., 2013; Groffen et al., 2013; Hahad et al., 2022; Hiles et al., 2015; Hoveling, Liefbroer, Schweren, Bültmann, & Smidt, 2022; Isensee, Wittchen, Stein, Höfler, & Lieb, 2003; Jackson et al., 2019; Kang & Lee, 2010; Kendler, Lönn, Sundquist, & Sundquist, 2015; Kim, Kim, Lim, & Kim, 2022; King, Jones, Petersen, Hamilton, & Nazareth, 2021; Korhonen, Ranjit, Tuulio-Henriksson, & Kaprio, 2017; Lam et al., 2005; Leung, Gartner, Hall, Lucke, & Dobson, 2012; Luijendijk, Stricker, Hofman, Witteman, & Tiemeier, 2008; Manrique-Garcia, Zammit, Dalman, Hemmingsson, & Allebeck, 2012; Meng et al., 2017; Monroe, McDowell, Kenny, & Herring, 2021; Monshouwer, ten Have, de Graaf, Blankers, & van Laar, 2021; Murphy et al., 2003; Mustonen et al., 2018a, 2018b, 2021; Najafipour et al., 2021; Okkenhaug, Tanem, Myklebust, Gjervan, & Johansen, 2018; Park, 2009; Paton, Kessler, & Kandel, 1977; Raffetti, Donato, Forsell, & Galanti, 2019; Ren et al., 2021; Rognli, Bramness, & von Soest, 2020; Rudaz et al., 2017; Sánchez-Villegas et al., 2021; Storeng, Sund, & Krokstad, 2020; Tanaka, Sasazawa, Suzuki, Nakazawa, & Koyama, 2011; Tomita & Manuel, 2020; Tsai, Chi, & Wang, 2013; Van Laar, Van Dorsselaer, Monshouwer, & De Graaf, 2007; van Os et al., 2002; Weiser et al., 2004; Werneck et al., 2022; Weyerer et al., 2013; Zammit, Allebeck, Andreasson, Lundberg, & Lewis, 2002; Zammit et al., 2003; Zhang, Woud, Becker, & Margraf, 2018; Zimmerman, Mast, Miles, & Markides, 2009; Zvolensky et al., 2008) of which 59 were included in the primary meta-analyses of adjusted estimates. No eligible studies of cannabis-tobacco co-use were identified.

### Study characteristics

Studies included in the primary meta-analyses consisted of 1 733 679 participants at risk of incident outcomes. Follow-up length ranged from 6 months to 63 years. Exposures were measured according to heaviness (e.g. cigarettes per day; *k* = 28) or status of use (e.g. current use; *k* = 31). Outcomes were assessed using symptom-based scales (*k* = 21), interviews (*k* = 18), registry codes (*k* = 14), self-reported treatment/diagnosis (*k* = 2), and composites (*k* = 4). Study characteristics are presented in the online Supplementary materials (eTables 2–7).

### Meta-analysis

**Figure 1.**
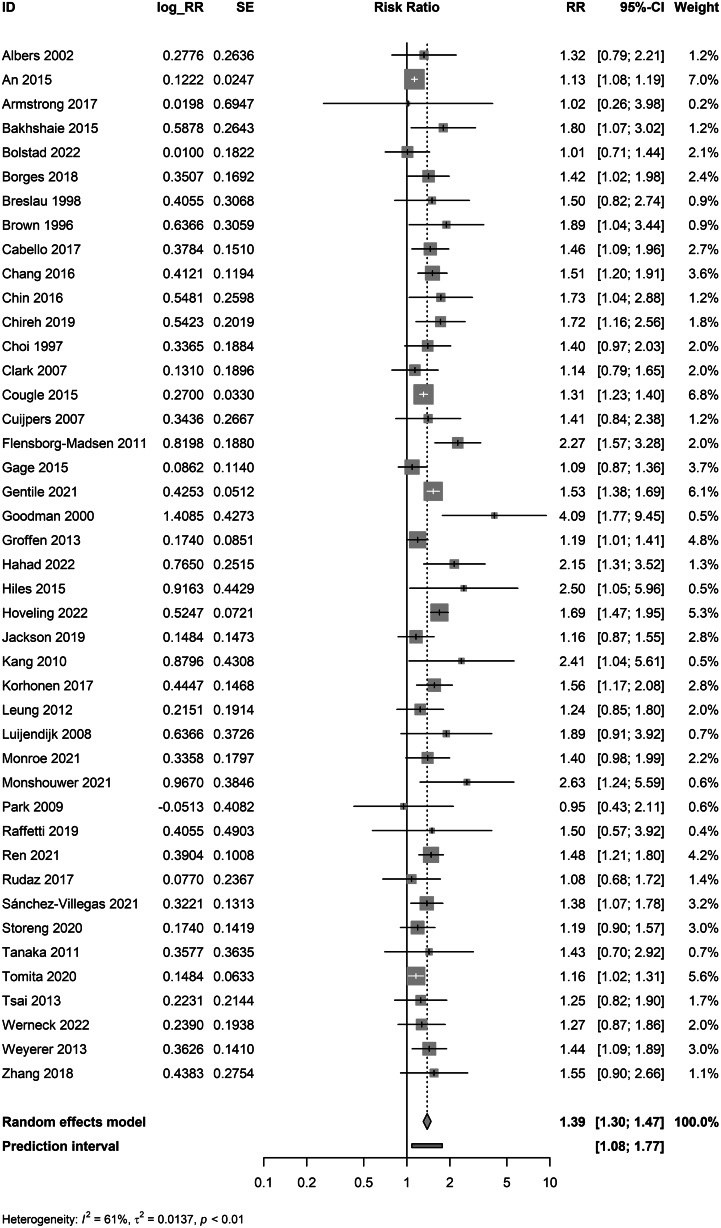
Meta-analysis of adjusted associations of tobacco use and mood disorders.

Tobacco use was associated with incident mood disorders (*K* = 43; RR: 1.39, 95% CI 1.30–1.47; *I*^2^ = 61.2%; *τ*^2^ = 0.014; PI: 1.08–1.77; Fig. 1) (Albers & Biener, 2002; An & Xiang, 2015; Armstrong et al., 2017; Bakhshaie et al., 2015; Bolstad et al., 2022; Borges et al., 2018; Breslau et al., 1998; Brown et al., 1996; Cabello et al., 2017; Chang et al., 2016; Chin et al., 2016; Chireh & D'Arcy, 2019; Choi et al., 1997; Clark et al., 2007; Cougle et al., 2015; Cuijpers et al., 2007; Flensborg-Madsen et al., 2011; Gage et al., 2015; Gentile et al., 2021; Goodman & Capitman, 2000; Groffen et al., 2013; Hahad et al., 2022; Hiles et al., 2015; Hoveling et al., 2022; Jackson et al., 2019; Kang & Lee, 2010; Korhonen et al., 2017; Leung et al., 2012; Luijendijk et al., 2008; Monroe et al., 2021; Monshouwer et al., 2021; Park, 2009; Raffetti et al., 2019; Ren et al., 2021; Rudaz et al., 2017; Sánchez-Villegas et al., 2021; Storeng et al., 2020; Tanaka et al., 2011; Tomita & Manuel, 2020; Tsai et al., 2013; Werneck et al., 2022; Weyerer et al., 2013; Zhang et al., 2018).

Exclusion of outliers (An & Xiang, 2015; Flensborg-Madsen et al., 2011; Goodman & Capitman, 2000), produced similar results (*K* = 40; RR: 1.38, 95% CI 1.31–1.45). Pooled unadjusted studies yielded a larger estimate (*K* = 41; RR: 1.47, 95% CI 1.34–1.60; online Supplementary eFig. 2).

**Figure 2.**
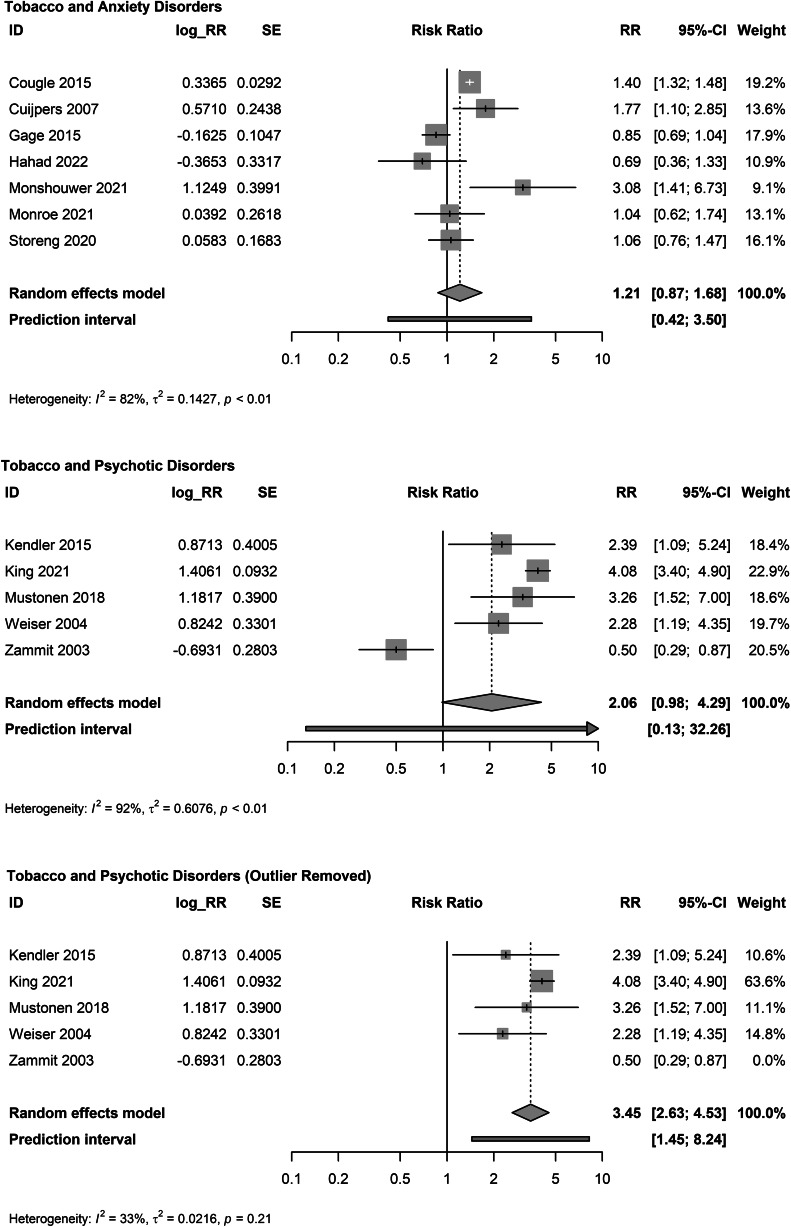
Meta-analyses of adjusted associations of tobacco use and anxiety and psychotic disorders.

Tobacco use was not associated with incident anxiety disorders (*K* = 7; RR: 1.21, 95% CI 0.87–1.68; *I*^2^ = 82.2%; *τ*^2^ = 0.143; PI: 0.42–3.50; Fig. 2) (Cougle et al., 2015; Cuijpers et al., 2007; Gage et al., 2015; Hahad et al., 2022; Monroe et al., 2021; Monshouwer et al., 2021; Storeng et al., 2020). There were no identified outliers. Pooled unadjusted studies yielded a larger estimate (*K* = 8; RR: 1.60, 95% CI 1.10–2.32; online Supplementary eFig. 3).

Tobacco use was not associated with incident psychotic disorders (*K* = 5; RR: 2.06, 95% CI 0.98–4.29; *I*^2^ = 92.3%; *τ*^2^ = 0.608; PI: 0.13–32.26 (Kendler et al., 2015; King et al., 2021; Mustonen et al., 2018a; Weiser et al., 2004; Zammit et al., 2003). Exclusion of one outlier (Zammit et al., 2003) yielded a larger pooled estimate with CIs that did not include the null (*K* = 4; RR: 3.45, 95% CI 2.63–4.53). As outlier identification was exploratory, pooled results with and without the outlier excluded are presented (Fig. 2). Pooled unadjusted studies yielded a larger estimate (*K* = 5; RR: 3.12, 95% CI 1.67–5.81; online Supplementary eFig. 4).

**Figure 3.**
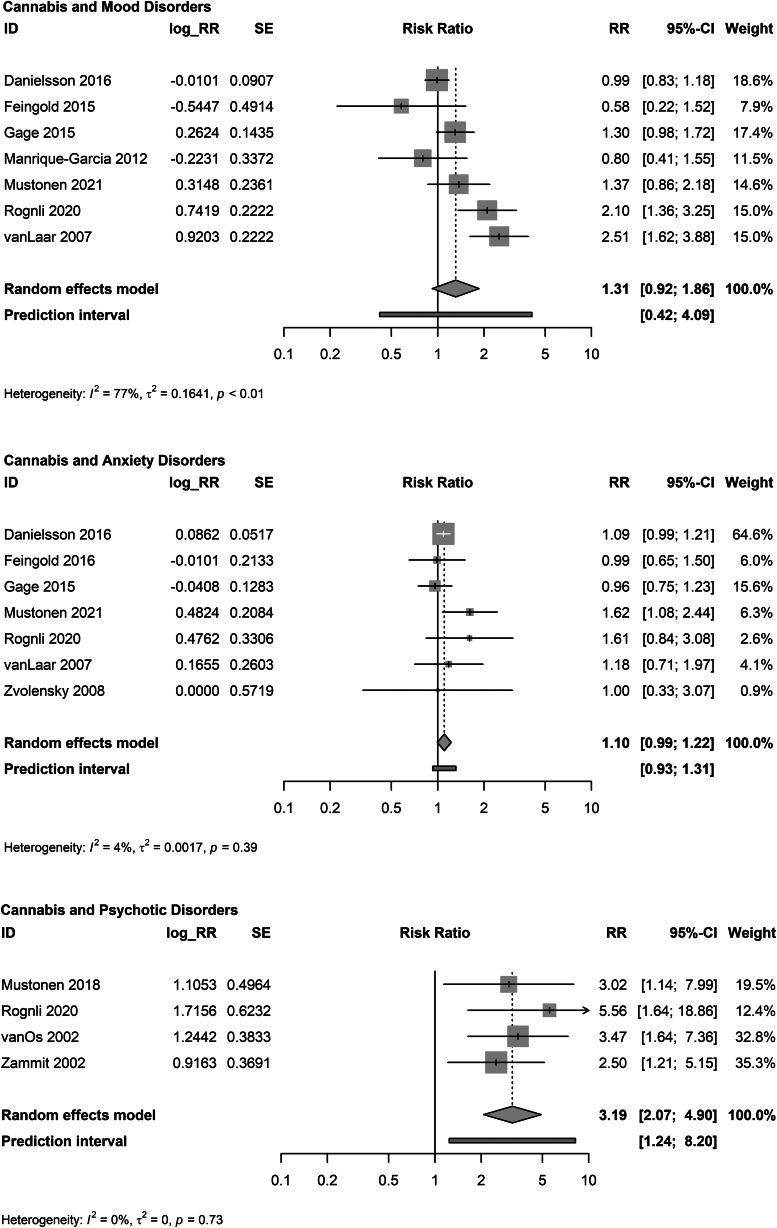
Meta-analyses of adjusted associations of cannabis use and mood, anxiety and psychotic disorders.

Cannabis use was not associated with incident mood disorders (*K* = 7; RR: 1.31, 95% CI 0.92–1.86; *I*^2^ = 77.0%; *τ*^2^ = 0.164; PI: 0.42–4.09; Fig. 3) (Danielsson et al., 2016; Feingold et al., 2015; Gage et al., 2015; Manrique-Garcia et al., 2012; Mustonen et al., 2021; Rognli et al., 2020; Van Laar et al., 2007). There were no identified outliers. Pooled unadjusted studies yielded a larger estimate (*K* = 7; RR: 1.47, 95% CI 1.19–1.81; online Supplementary eFig. 5).

Cannabis use was not associated with incident anxiety disorders (*K* = 7; RR: 1.10, 95% CI 0.99–1.22; *I*^2^ = 4.4%; *τ*^2^ = 0.002; PI: 0.93–1.31; Fig. 3) (Danielsson et al., 2016; Feingold, Weiser, Rehm, & Lev-Ran, 2016; Gage et al., 2015; Mustonen et al., 2021; Rognli et al., 2020; Van Laar et al., 2007; Zvolensky et al., 2008). There were no identified outliers. Pooled unadjusted studies yielded a larger estimate (*K* = 6; RR: 1.51, 95% CI 1.20–1.89; online Supplementary eFig. 6).

Cannabis use was associated with incident psychotic disorders (*K* = 4; RR: 3.19, 95% CI 2.07–4.90; *I*^2^ = 0%; *τ*^2^ = 0.00; PI: 1.24–8.20; Fig. 3) (Mustonen et al., 2018b; Rognli et al., 2020; van Os et al., 2002; Zammit et al., 2002). There were no identified outliers. Pooled unadjusted studies yielded a larger estimate (*K* = 3; RR: 4.68, 95% CI 3.30–6.64; online Supplementary eFig. 7).

### Quality assessment

Of the 59 studies included in the adjusted meta-analyses, roughly one-quarter of studies (27%) were rated as high quality (i.e. lower risk of bias; online Supplementary eTable 8), with an overall mean score of 7.35 (s.d. 1.01). The proportion of high-quality studies differed by analysis (online Supplementary eTable 9). Many studies (58%) were marked down due to high attrition or insufficient information about loss to follow-up (e.g. differential attrition), and 41% of studies were marked down for ‘comparability’ (i.e. confounding bias).

### Subgroup and sensitivity analyses

Using the confounder matrix, most studies had multiple confounding constructs rated as inadequately adjusted for (Table 2, online Supplementary eTables 10–15, eFigs 8–13). Sociodemographic factors (e.g. age, sex) were generally well-adjusted for across all analyses. Psychological factors (e.g. loneliness, adverse childhood experiences [ACEs]) and psychiatric comorbidity were generally insufficiently controlled for with lower rates of adequate adjustment, particularly in tobacco and mood studies. There were differences in adjustment patterns across analyses, for example a higher proportion of cannabis studies (e.g. 100% of cannabis and mood studies) were rated as adequately adjusting for other substance use (i.e. alcohol use, illicit drug use), whereas co-use was more comprehensively adjusted for in tobacco studies as none of the included cannabis studies adjusted for confounding via co-administration of tobacco. Adjustment for other substance use by subconstructs (i.e. alcohol use, illicit drug use) is available in online Supplementary eTables 10–15. All cannabis studies were rated as inadequate adjustment for other lifestyle factors (e.g. physical activity, diet), with evidence of more adjustment in studies of tobacco and mood and anxiety disorders. Percentages of studies by adjustment rating for the different constructs are reported in Table 2, alongside median *E*-values for study point estimates and CIs. Median *E*-values for the point estimate ranged from 1.40 to 5.95.

**Table 2.** *E*-value and confounder matrix summary

	Confounder summary matrix^a^							Median *E*-value^b^	
	Co-use	Other substance use	Psychiatric comorbidity	Socio-demographic factors	Socio-economic status	Psychological factors	Other lifestyle factors	Point estimate	CI
Tobacco and mood (*K* = 43)									
Adequate	63%	49%	7%	65%	47%	26%	44%	2.21	1.16
Concerns	0%	23%	19%	30%	33%	30%	21%		
Inadequate	37%	28%	74%	5%	21%	44%	35%		
Tobacco and anxiety (*K* = 7)									
Adequate	86%	57%	29%	86%	57%	43%	57%	2.15	1.00
Concerns	0%	14%	43%	14%	43%	29%	14%		
Inadequate	14%	29%	29%	0%	0%	29%	29%		
Tobacco and psychosis (*K* = 5)									
Adequate	60%	20%	40%	20%	80%	40%	0%	4.21	1.67
Concerns	0%	40%	0%	40%	0%	20%	0%		
Inadequate	40%	40%	60%	40%	20%	40%	100%		
Cannabis and mood (*K* = 7)									
Adequate	0%	100%	43%	86%	43%	57%	0%	2.08	1.00
Concerns	57%	0%	57%	14%	43%	29%	0%		
Inadequate	43%	0%	0%	0%	14%	14%	100%		
Cannabis and anxiety (*K* = 7)									
Adequate	0%	86%	43%	71%	43%	43%	0%	1.40	1.00
Concerns	57%	14%	43%	14%	29%	29%	0%		
Inadequate	43%	0%	14%	14%	29%	29%	100%		
Cannabis and psychosis (*K* = 4)									
Adequate	0%	50%	50%	75%	0%	25%	0%	5.95	1.20
Concerns	75%	25%	0%	0%	50%	75%	0%		
Inadequate	25%	25%	50%	25%	50%	0%	100%		

*Note.*^a^Percentages denote the proportion of studies in the adjusted meta-analyses that were rated as ‘adequate’, ‘some concerns’, or ‘inadequate’ for the different constructs and assessment criteria for different constructs is detailed in online Supplementary eMethods 8; briefly: co-use (i.e. adjustment for cannabis/tobacco use); other substance use (i.e. adjustment for alcohol use and illicit drug use); psychiatric comorbidity (i.e. adjustment for other mental health condition(s) at baseline); sociodemographic factors (i.e. adjustment for age, sex and ethnicity, urbanicity, or marital status); socioeconomic status (i.e. adjustment for combination of indicators like education and income, or index of socioeconomic status); psychological factors (i.e. adjustment for two factors from varied list including loneliness, adverse childhood experiences, IQ, and stressful life events); other lifestyle factors (i.e. adjustment for two factors from physical activity, health conditions, adiposity, and diet).

b The *E*-value represents the minimum strength of association, on the RR scale, that an unmeasured confounder would need to have with both the exposure and the outcome to fully explain away a specific exposure–outcome association, conditional on the measured covariates. This interpretation applies to the point estimate and the lower CI. Generally, a larger *E*-value implies that considerable unmeasured confounding would be needed to explain away an effect estimate. Generally, a smaller *E*-value implies little unmeasured confounding would be needed to explain away an effect estimate. For more information see online Supplementary eMethods 6.

As an example, the median *E*-value of the point estimate of the association of tobacco use and incident mood disorders was 2.21. This suggests if an omitted set of unmeasured confounders had an RR of 2.21 on both tobacco use and mood disorders, conditional on measured covariates, the association between tobacco use and mood disorders may be reduced to the null in half the studies. The same approach applies to the median *E*-value of the CI, i.e. if an omitted set of unmeasured confounders had an RR of 1.16 on both tobacco use and mood disorders, conditional on measured covariates, the association between tobacco use and mood disorders may be reduced to the null in half the studies.

Doi plots and LFK indices (online Supplementary eFigs 14–19) indicated major asymmetry in tobacco use and mood (LFK = 4.12) and psychotic disorders (LFK = −3.86). The remaining exposure–outcome analyses were indicated to have minor asymmetry (LFK = −1.68 to 1.89; Table 3), except for cannabis use and mood disorders (LFK = −0.59).

**Table 3.** LFK index and asymmetry rating for primary meta-analyses

Exposure	Outcome	*K*	LFK index score	Asymmetry rating^a^
Tobacco	Mood	43	4.12	Major
Tobacco	Anxiety	7	−1.68	Minor
Tobacco	Psychosis	5	−3.86	Major
Cannabis	Mood	7	−0.59	No asymmetry
Cannabis	Anxiety	7	1.89	Minor
Cannabis	Psychosis	4	1.73	Minor

*Note. K*, number of studies included in meta-analysis.

a LFK index scores of ±1, between ±1 and ±2, or ±2 indicate ‘no asymmetry’, ‘minor asymmetry’, and ‘major asymmetry’ respectively.

Subgroup and meta-regression analyses were only performed for tobacco use and mood disorders due to low study numbers (*K* < 10) in other meta-analyses. Results were examined across age groups, follow-up length, sample size, study quality, confounding adjustment, and exposure/outcome types. The analyses did not support evidence of subgroup effects (online Supplementary eTables 16 and 17), including analyses by adequate adjustment for co-use and overall confounding adjustment. However, a far smaller number of studies contributed to some subgroups and there is substantial heterogeneity across the included studies, meaning results should be interpreted with caution (Richardson, Garner, & Donegan, 2019).

## Discussion

To our knowledge, this is the first systematic review and meta-analysis examining the association of tobacco use, cannabis use, and incident mental illness that has undertaken a comprehensive assessment of the influence of confounding bias. We found evidence for associations of tobacco and incident mood and psychotic disorders, and for cannabis and incident psychotic disorders. Our review includes the first meta-analysis of the longitudinal association between tobacco use and incident anxiety disorders and addresses limitations of previous reviews which have considered evidence for both substances and psychological outcomes.

Accurately understanding the causal effects of substance use on mental illness is crucial to informing effective evidence-based public health policies (Taylor & Treur, 2023). Results from this review are based on observational evidence and cannot in isolation be considered proof of causality. However, this study contributes toward a wider, growing body of evidence across multiple study designs (e.g. Mendelian randomization [MR], smoking cessation trials) that these substances have a causal role in development of psychotic disorders, and tobacco use in mood disorders (Firth, Wootton, Sawyer, & Taylor, 2023; Ganesh & D'Souza, 2022; Munafò, 2022).

We did not find compelling evidence to suggest tobacco use is associated with incident anxiety disorders. Previous narrative syntheses report mixed evidence of associations between tobacco use and later anxiety (Fluharty et al., 2017; Stevenson et al., 2022). The effect size observed in the analysis of tobacco use and mood disorders is consistent with previous meta-analyses (Chaiton et al., 2009; Chaplin et al., 2023; Esmaeelzadeh et al., 2018; Luger et al., 2014). Although there was considerable methodological heterogeneity present across studies, tests for subgroup differences did not indicate any significant differences. Importantly, non-significant subgroup tests do not automatically imply equivalent results. If there is substantial between-study heterogeneity within the subgroup this will decrease the precision of the pooled effect, and mean CIs are more likely to overlap such that specific subgroup effects may be affected by other sources of heterogeneity across the review (Harrer, Cuijpers, Furukawa, & Ebert, 2021; Richardson et al., 2019).

Our analyses of cannabis use and subsequent mood and anxiety disorders did not support evidence of an increased risk in the cannabis use *v.* non-use groups. Several previous meta-analyses have reported mixed evidence of associations between cannabis use and anxiety symptoms or disorder (Hall, Leung, & Lynskey, 2020), and multiple meta-analyses of prospective studies report a modest association between cannabis use and depressive symptoms or disorder (Hall et al., 2020). Three previous meta-analyses of prospective studies, adjusting for baseline depression, report modest associations (odds ratio [OR] range: 1.17–1.37; Esmaeelzadeh et al., 2018; Gobbi et al., 2019; Lev-Ran et al., 2014) between cannabis use and subsequent depression. It is possible that examining incident outcomes (*v*. statistical adjustment) could explain the discrepancy in findings, but may also relate to other differences in review content (e.g. adolescents only, more studies). Recent reviews focusing on studies of cannabis frequency and potency suggest that more frequent use (Robinson et al., 2023) and higher-potency cannabis (Petrilli et al., 2022) poses greater risk. However, due to limited study numbers and measurements, it was not possible to investigate this.

In line with other meta-analyses, this study reported evidence of a strong association between both substances and psychotic disorders (Gurillo et al., 2015; Hunter et al., 2020; Marconi et al., 2016; Moore et al., 2007; Myles et al., 2012; Robinson et al., 2023). Considerable uncertainty regarding the size of the association was indicated by CIs and PIs. ‘Noisy’ effect estimates are common in the case of rare outcomes, due to lower statistical power. Pooling these effects in a meta-analysis can yield more precise estimates, but this review included few studies. This is likely related to the exclusion of traditional case-control designs. Although well suited to the study of rare outcomes, they are at increased risk of recall bias and reverse causation (Rothman, Lash, VanderWeele, & Haneuse, 2021). Lack of prospective research in this area has been highlighted (Quigley & MacCabe, 2019; Sideli, Quigley, La Cascia, & Murray, 2020).

We did not identify any eligible studies of cannabis–tobacco co-use. Assuming causality, dual use may place consumers at a higher risk of developing a mental health condition than independent use of either substance. There is a handful of cross-sectional studies which indicate people who co-use have a higher prevalence of mental health disorders (Hindocha, Brose, Walsh, & Cheeseman, 2020; Peters, Schwartz, Wang, O'Grady, & Blanco, 2014) and levels of psychological distress (Wang, Yao, Sung, & Max, 2022). Some longitudinal evidence suggests co-use is associated with greater mental health symptoms (Tucker et al., 2019), but prospective evidence is lacking.

While adjustment for other substance use (i.e. alcohol use, illicit drug use) was often applied, adjustment for co-use was mixed and none of the included cannabis use studies measured or adjusted for tobacco co-administration. Although degree of confounding bias will differ at a population-level across countries due to international differences in co-administration prevalence (e.g. Europe *v.* America; Hindocha, Freeman, Ferris, Lynskey, & Winstock, 2016), this remains an important source of information to collect and adjust for within individual cohorts as people who co-administer cannabis with tobacco (e.g. blunts, spliffs) will frequently self-report to be non-smokers (Hindocha & McClure, 2020).

Analyses of small-study effects suggested possible risk of publication bias, with evidence of asymmetry for most meta-analyses. As such, pooled estimates may misrepresent the ‘true’ association. However, asymmetry can be driven by multiple factors (e.g. methodological heterogeneity) and may not represent publication bias (Sterne & Harbord, 2004). Furthermore, although Doi plots have advantages over traditional funnel plots in detecting asymmetry with few studies (*K* < 10), they may still misrepresent asymmetry (Furuya-Kanamori et al., 2018).

*E*-values and confounder matrix assessment suggested that many studies were at risk of confounding bias. Studies often inadequately adjusted for key confounding variables (e.g. ACEs). Previous reviews of these exposures have demonstrated moderate-strong adjusted associations with substance use and mental health outcomes (e.g. ACEs: OR_Smoking_ 2.82, OR_Depression_ 4.40; Hughes et al., 2017). Furthermore, none of the study estimates adjusted for genetic vulnerability which alternative study designs (e.g. familial-based designs) suggest may play a substantial role in the observed associations (Barkhuizen, Taylor, Freeman, & Ronald, 2019; Ranjit et al., 2019; Schaefer et al., 2021). *E*-values must be interpreted considering some key assumptions and limitations (VanderWeele, 2022; VanderWeele et al., 2019). Importantly, adjustment for some measured covariates (e.g. socioeconomic status) likely reduces bias from some unmeasured confounding (e.g. ACEs) due to associations between these constructs. The *E*-value is also conservative (i.e. overestimates bias), insofar as it assumes the distribution of the unmeasured confounder(s) is as unfavorable as possible (VanderWeele et al., 2019). Nonetheless, the smaller *E*-values observed for some estimates (i.e. tobacco/mood) in the presence of multiple unmeasured confounders suggests that the pooled estimates likely overestimate the effect size.

Although unmeasured confounding was a focus of this review, many studies were also limited by inadequate description of attrition or individual-level missing data and few used methods to account for this (e.g. multiple imputation). This contradicts recommendations by relevant reporting guidelines (e.g. STROBE; Vandenbroucke et al., 2007), and hinders assessment of selection bias. Future studies aiming to explore causal effects must provide more detailed descriptions of missing data and apply appropriate methods to reduce bias (VanderWeele, 2021). Furthermore, although we focused on incident outcomes in prospective studies this does not exclude risk of bias from reverse causation. Many mental disorders do not have discrete onsets, and there are challenges to accurately defining incidence including subthreshold or prodromal symptoms at baseline (Patten, 2021) and diagnostic lag (e.g. in studies using registry data). As such, to support the identification of causal effects, there is the need for further research focusing on addressing and exploring the biases that arise in conventional observational studies.

MR is one such method, which uses genetic variation as an instrumental variable for an exposure to estimate causal effects that are more robust to reverse causality and confounding bias (Davies, Holmes, & Smith, 2018). Reviews of MR studies investigating substance use and mental health suggest evidence to support a bi-directional, increasing relationship between smoking and depression, bipolar disorder and schizophrenia (Treur, Munafò, Logtenberg, Wiers, & Verweij, 2021). Evidence regarding cannabis use and mental health is less conclusive, which may relate to historical lack of frequency instruments (Hines, Treur, Jones, Sallis, & Munafò, 2020; Treur et al., 2021). However, MR is ‘far from a silver bullet’ (Wootton, Jones, & Sallis, 2022) with limitations to be addressed through more advanced methods (e.g. multivariable MR), additional sensitivity tests (e.g. residual population stratification), and incorporation into planned triangulation frameworks (Hammerton & Munafò, 2021), including triangulation with carefully planned longitudinal cohort analyses (Hammerton & Munafò, 2021; Treur et al., 2021). Widespread adoption of DAGs when selecting secondary data sources may yield insights as to whether research questions are feasibly explored within datasets (VanderWeele et al., 2019). Alongside the need for well-controlled longitudinal studies, more evidence using alternative study designs is required as meta-analyses of the same study design may amplify inherent biases.

### Limitations

Several important limitations need to be considered. All studies used self-report to define exposure status. This is not unusual in large, population-based cohort studies but will result in measurement error that can bias effect estimates in the case of both differential and non-differential misclassification. Similarly, we included studies which used symptom-based scales, self-reported diagnosis, and resource access (e.g. medication) which will introduce further measurement error. Most studies were based in high-income countries and to reduce sources of heterogeneity we restricted the review to include a specific type of study design (i.e. prospective, incident outcomes) conducted in general population samples. This does not capture all evidence regarding the link between substance use and mental illness, such as evidence that cannabis and tobacco use may impair treatment outcomes in people with mental health conditions (Asharani & Subramaniam, 2022; Reid & Bhattacharyya, 2019; Sideli et al., 2020; Tourjman et al., 2023) and increased risk in people with underlying risk factors (e.g. ultra-high risk for psychosis; Andreou et al., 2023). Understanding the causal effect of these substances on mental health in vulnerable groups is essential for designing targeted interventions and addressing existing health inequalities. The number of studies included in most meta-analyses was low and prevented planned explorations of heterogeneity, which is recommended for syntheses of non-randomized studies (Egger, Higgins, & Smith, 2022). Finally, analyzing overarching diagnostic groups (e.g. mood disorders) may overlook relevant differences for individual disorders (e.g. bipolar disorder) which will be important to consider in exploring possible causal mechanisms (e.g. neuroadaptations in nicotinic pathways; Firth et al., 2023).

## Conclusion

This review and meta-analysis presents evidence for longitudinal associations between both substances and incident psychotic disorders, and tobacco use and incident mood disorders. In contrast to previous meta-analyses, there was no clear evidence to support an association between cannabis use and incident mood or anxiety disorders. Existing evidence across all outcomes was limited by inadequate adjustment for potential confounders. Future research should prioritize approaches supporting stronger causal inference, such as evidence triangulation.

## Supporting information

Burke et al. supplementary material Burke et al. supplementary material

## Data Availability

Data and R code for the analyses included in this study have been provided online at https://github.com/chloeeburke/tobcanmeta.

## Supplementary material

The supplementary material for this article can be found at https://doi.org/10.1017/S0033291724002587.
